# Herpes Simplex Pneumonia in an Immunocompetent Patient With Progression to Organizing Pneumonia

**DOI:** 10.1177/2324709614530560

**Published:** 2014-04-09

**Authors:** Brooke Mills, Atul Ratra, Amal El-Bakush, Shrinivas Kambali, Kenneth Nugent

**Affiliations:** 1 Texas Tech University Health Sciences Center, Lubbock, TX, USA

**Keywords:** herpes simplex, acute respiratory failure, organizing pneumonia

## Abstract

*Background*. Organizing pneumonia is an uncommon diffuse interstitial lung disease that affects the terminal and respiratory bronchioles, alveolar ducts, and alveoli. Most cases are idiopathic, but some are associated with infections. We present an uncommon case of organizing pneumonia associated with herpes simplex virus-1 (HSV-1). *Case*. A 39-year-old man with hypertension presented with dyspnea, fever, and productive cough for 2 weeks. He was treated for 5 days for acute bronchitis as an outpatient with no improvement. His examination revealed mild respiratory distress, O_2_ saturation 92% on room air, and right sided crackles. Labs included a white blood cell count of 19 300/µL. His chest x-ray showed bilateral infiltrates greater on the right. Bronchoalveolar lavage was positive for HSV-1; transbronchial biopsies showed focal pneumonitis with plentiful intra-alveolar macrophages. His respiratory status progressively deteriorated, and he was intubated for mechanical ventilation. He received 10 days of intravenous (IV) antibiotics and 14 days of IV acyclovir. He was readmitted 10 days later with worsening symptoms and was intubated for respiratory failure. His CT chest showed diffuse, patchy consolidation of both lungs, right more than left. Open lung biopsy showed extensive organizing pneumonia, diffuse alveolar damage, intra-alveolar macrophages, and pleural fibrosis; he was treated with IV corticosteroids. He was extubated after 10 days; within 2 weeks his chest x-ray was markedly improved. *Discussion*. Organizing pneumonia is usually idiopathic; infection is one of the secondary causes. To our knowledge this is only the second reported case associated with HSV. This association may have important pathogenic and therapeutic implications.

## Introduction

Herpes simplex pneumonia occasionally occurs in immunocompetent patients. This may develop as a primary infection or as a reactivation of latent infection during an acute illness, especially respiratory failure. These infections may have a role in the pathogenesis of respiratory failure in acute respiratory distress syndrome (ARDS) patients, but this is unclear. We report a case of herpes pneumonia in a patient with acute respiratory failure who subsequently developed organizing pneumonia.

## Case

A 39-year-old man with no significant past medical history who works as an oil well driller presented to the emergency department (ED) with a 3-day history of significant shortness of breath and a productive cough. Two weeks prior, he had presented to the ED with symptoms of shortness of breath and productive cough and was treated with a onetime dose of azithromycin 500 mg, a 4-day course of azithromycin 250 mg once daily, a 5-day course of prednisone 10 mg 4 times daily, and albuterol 17 two puffs 4 times daily. He remained short of breath at rest and with activity. He complained of a productive cough with yellowish sputum and occasional streaks of blood. Pleuritic chest pain was present with coughing. He also had mild epigastric abdominal pain, nausea, and vomiting for the previous 7 to 10 days with no aggravating or alleviating factors. He denied any diarrhea or constipation. He denied any recent fever but had recently noticed significant diaphoresis. He denied any recent sick contacts, recent travel, and any history of asthma or tuberculosis. He had smoked 1 pack of cigarettes per day for an undocumented amount of time until 1 month previously when he quit.

His vital signs were as follows: blood pressure 167/104 mm Hg, heart rate 114 beats per minute, respiratory rate 24 breaths/min, temperature 98.7°F, O_2_ saturation 92% on room air, and body mass index 47.5 kg/m^2^. On examination he was mildly dyspneic and diaphoretic. He had no oral lesions. He had diffuse rhonchi bilaterally on inspiration and expiration and crackles on the right. His abdomen was soft, nontender, and nondistended with active bowel sounds.

His labs were as follows: white blood cell (WBC) 19,300/µL, hemoglobin 12.1 g/dL, hematocrit 36.4%, normal electrolytes, creatinine 1 mg/dL, glucose 117 mg/dL, total bilirubin 2.2 mg/dL, albumin 3.1 g/dL, alanine aminotransferase (ALT) 63 IU/L, and aspartate aminotransferase (AST) 38 IU/L. Cardiac enzymes were negative. Hepatitis panel was negative. His chest x-ray revealed bilateral lung infiltrates. The chest computed tomography (CT) showed no evidence of pulmonary thromboembolism and extensive patchy ground glass opacities in both lungs consistent with possible pulmonary edema, ARDS, or severe pneumonitis. The patient was admitted to the hospital and started on levofloxacin 750 mg once daily, piperacillin/tazobactam 3.375 g 3 times daily, vancomycin 1.25 g twice daily, and methylprednisolone 80 mg 4 times daily. The methylprednisolone dose and frequency was tapered down to 40 mg once daily over a 7-day period before it was discontinued.

His blood cultures showed no growth after 5 days, and his sputum culture grew 2+ normal upper respiratory flora and <1+ *Staphylococcus aureus*, resistant only to penicillin. Studies for *Legionella pneumophila*, HIV, and *Pneumocystis jiroveci* were negative. Five days after admission, he underwent bronchoscopy with bronchial alveolar lavage (BAL) from the right upper and lower lobes. The bronchoscopist found inflamed mucosa throughout the tracheobronchial tree, which was worse on the right side, thin mucoid secretions, especially in the right upper lobe, and no focal mucosal lesions. Transbronchial biopsies showed focal pneumonitis with plentiful intra-alveolar macrophages. His respiratory virus immunofluorescent assays (IFA) from the BAL were negative for adenovirus, influenza types A and B, respiratory syncytial virus, metapneumovirus, and parainfluenza virus types 1, 2, and 3. Direct fluorescent antibody (DFA) for PCP and CMV were negative. Herpes simplex DFA screening was positive for herpes simplex virus 1 (HSV-1) both from bronchial washings and both BAL specimens using the D3 DFA Herpes Simplex virus identification and typing kit (Diagnostic Hybrids, Athens, OH). (The kit’s positive and negative percent agreements for HSV-1 were 92.7% to 100% and 98.9% to 100%, respectively. Positive and negative percent agreements are used instead of sensitivity and specificity when standard references are not available to form the results. The term percentage therefore measures the percent agreement with a reference that is not standard rather than the accuracy of the study.^1^) After 7 days, no herpes simplex virus was isolated on culture. No yeast or fungal elements were seen; aerobic cultures were negative on BAL and bronchial washing. Anaerobic cultures grew <1+ *Staphylococcus saccharolyticus* and <1+ *Campylobacter gracilis*. BAL fluid analysis showed a red blood cell count of 4232/mm^3^ and a WBC count of 224/mm^3^. The WBC differential was 85% neutrophils, 2% lymphocytes, 2% monocytes, 2% eosinophils, 8% macrophages, and 1% mesothelial cells. A 14-day course of acyclovir 1.3 g 3 times daily was started for herpes infection. Piperacillin/tazobactam 3.375 g twice daily, levofloxacin 750 mg once daily, and methylprednisolone 40 mg twice daily were continued. Vancomycin was discontinued.

**Figure 1.**
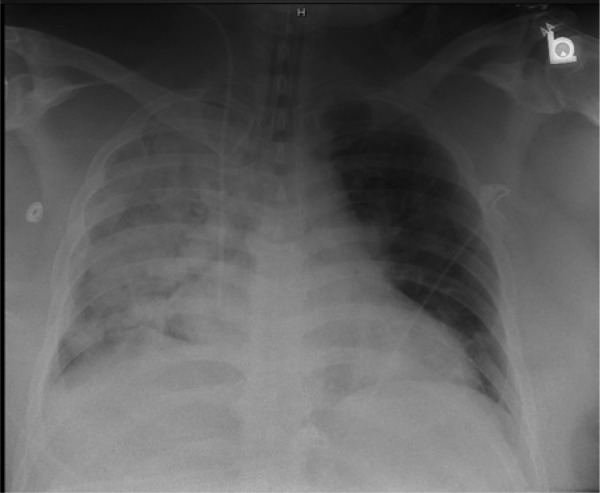
Day of initial intubation. Right alveolar infiltrate.

Six days after admission, the patient’s breathing became labored and tachypneic, and he was intubated due to respiratory failure (Figure 1). His PPD was negative, and his CD4 and CD8 counts were 613/µL (normal range = 347-872/µL) and 380/µL (normal range = 224-872/µL), respectively. Piperacillin/tazobactam and levofloxacin were discontinued on day 9, and methylprednisolone was discontinued on day 10. The patient was intubated for a total of 9 days. Two days after extubation, he was discharged.

**Figure 2.**
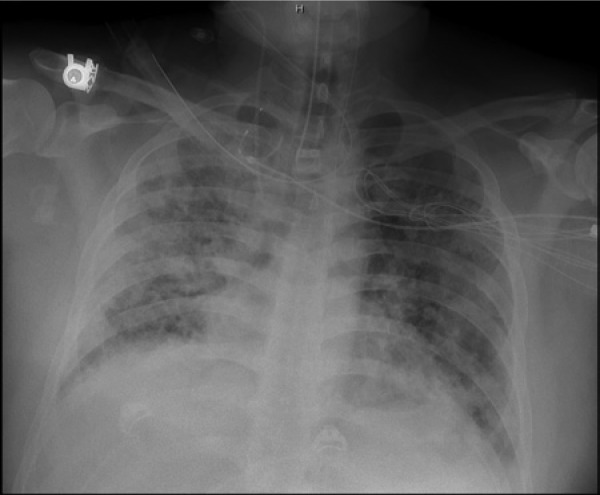
Day of open lung biopsy. Bilateral diffuse infiltrates.

**Figure 3.**
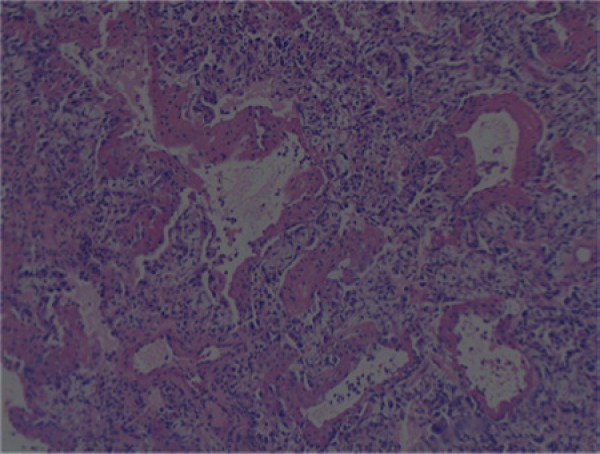
Right lung wedge biopsy. Hyaline membranes in alveolar spaces.

**Figure 4.**
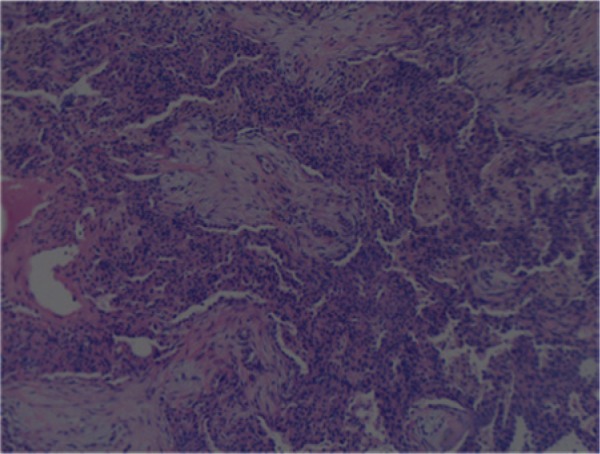
Right lung wedge biopsy. Fibrous plugs in bronchioles and alveolar spaces.

Twelve days after discharge, the patient returned with increasing shortness of breath and bloody sputum for the previous 3 days. His vital signs were as follows: blood pressure 142/91 mm Hg, heart rate 99 beats per minute, and respiratory rate 24 breaths/min. His physical examination was significant for decreased breath sounds especially on the right side and bilateral crackles with occasional wheezing. His labs were as follows: WBC 13,300/µL, hemoglobin 11.3 g/dL, hematocrit 35.4%, sodium 140 mmol/L, potassium 3.2 mmol/L, BUN 8 mg/dL, creatinine 0.8 mg/dL, glucose 117 mg/dL, total bilirubin 1.6 mg/dL, albumin 3.7 g/dL, ALT 23 IU/L, AST 17 IU/L. Cardiac enzymes were negative. A chest CT showed worsening consolidation of the lungs bilaterally, especially in the right lung. Sputum and blood cultures were negative. He was started on vancomycin 2 g 3 times daily, azithromycin 500 mg once daily, cefepime 1 g 3 times daily, and acyclovir 1.2 g 3 times daily but showed little improvement. He was intubated 4 days after admission for a total of 21 days (Figure 2). Ten days after admission he had an open lung right wedge biopsy that showed organizing pneumonia changes with interstitial pneumonitis (Figures 3 and 4). Pathology also showed diffuse alveolar damage with hyaline membranes, intra-alveolar macrophages, and pleural fibrosis. He was started on methylprednisolone 40 mg 4 times daily over the next 14 days and his oxygenation improved. Three days after extubation the patient left against medical advice (AMA). He presented in the internal medicine clinic for a follow-up appointment where he stated that he was admitted to a different hospital the same day he left AMA. He stayed at the other hospital for 2 weeks and was continued on prednisone. At the clinic he reported a cough but no dyspnea or limitations of activity. He was off prednisone.

## Discussion

Herpes pneumonia in an immunocompetent patient is rare. Our patient had an acute respiratory syndrome, diffuse patchy infiltrates on chest x-ray, worse on the right side, consistent with pneumonia, and negative fungal, bacterial, and viral studies on BAL fluid from 2 separate lobes. The diagnosis of herpes was based on the positive DFA from bronchial washings and BAL specimens. The negative herpes culture can be attributed to either a lab error or chance. Current preparation guidelines for HSV-1 cultures require refrigeration of the specimen at 4°C until inoculation.^2^ In this case the fluids were left at room temperature for approximately 3 hours before inoculation, possibly leading to viral death. Furthermore, the sensitivity and specificity of herpes cultures are 80.7% and 99%, respectively.^3^ Due to the low sensitivity of viral culture, false negative cultures do occur. The information available in this case does not support an alternative diagnosis.

HSV-1 usually infects the upper airway and causes gingivostomatitis and pharyngitis. The virus becomes latent in the trigeminal nerve ganglia and can reactivate to cause herpes labialis. Visceral herpes infection, including pneumonia, typically occurs in immunocompromised patients.^4^ The virus can reach the lower respiratory tract through aspiration of infected oropharyngeal secretions, through continuous extension of the infection below the vocal cords into the tracheobronchial tree, through reactivation of virus in vagal nerve ganglia, or through systemic dissemination to the lung from remote sites of infection.^5^ HSV-1 occurs relatively frequently in patients who require prolonged mechanical ventilation for acute respiratory failure.^6^ In many of these patients the herpes infection represents a marker of disease severity rather than an active viral infection which causes inflammation and disease.^7^ Luyt and coworkers reported that herpes bronchopneumonia developed in 20% of critically ill patients on ventilators for more than 5 days, and that these patients had worse outcomes (longer periods of mechanical ventilation and intensive care unit lengths of stay).^6^ Tuxen studied the effects of prophylactic acyclovir in patients requiring prolonged mechanical ventilation and demonstrated that this antiviral medication could reduce the frequency of herpes recovery from the lower respiratory tract but did not change outcomes.^8^ This study would suggest that, in general, herpes infections do not have an independent effect on prognosis and outcome. We identified 5 patients using PubMed searches who appeared to have primary herpes pneumonia at presentation and had significant clinical disease.^9-13^ These patients had not required prolonged mechanical ventilation before the infection was identified (Tables 1 and 2). Our patient presumably had a reactivation of herpes in either the upper respiratory tract with dissemination to the lower respiratory tract or had reactivation of HSV-1 in the lung from latent of viral reservoirs in the vagal nuclei. His bronchoscopy revealed diffuse erythema of airways, which might suggest that he had direct extension of infection from the upper airway. He had virus recovered from both the right upper and right lower lobes. He had no other bacterial or fungal or viral pathogens identified in bronchoalveolar lavage fluid and improved with acyclovir treatment. Serological studies could help identify a primary herpes infection but were not done in this case. However, this information would not alter our conclusion that this patient had herpes pneumonia.

**Table 1.** Herpes Pneumonitis in Immunocompetent Adults.

Reference No.	Case Summary	Authors’ Conclusion
9	30-Year-old woman with acute respiratory distress syndrome. Patient died after 2 weeks of antibiotic treatment. Postmortem studies led to the diagnosis of HSV pneumonia.	HSV DNA detection by PCR yields a quick and accurate way to diagnose potentially fatal HSV pneumonia infections.
10	33-Year-old man admitted with lower respiratory tract infection symptoms. Patient was initially treated with IV antibiotics but showed no improvement. Cytological studies led to the diagnosis of HSV pneumonia and successful treated with acyclovir.	HSV pneumonia must be considered in the evaluation of an immunocompetent patient presenting with a lower respiratory tract infection that is refractory to antibiotic therapy.
11	28-Year-old man on oral corticosteroids for acute asthma exacerbation developed worsening lower respiratory tract infection symptoms. Tracheal biopsy, PCR, and viral cultures revealed HSV-1 infection in the respiratory tract. Patient was successfully treated with antibiotics and acyclovir.	HSV-1 can cause acute tracheitis in immunocompetent individuals. HSV tracheitis can potentially facilitate transmission of bacteria into the lungs from the oropharynx through the aspiration of mucosal secretions and development of bacterial pneumonia.
12	18-Year-ole woman admitted with severe respiratory distress and HSV-1 outbreak on her lip. Patient was treated with broad spectrum antibiotics but showed no improvement. Patient was diagnosed with HSV-1 pneumonia based on cytological and serum studies and successfully treated with acyclovir.	Primary HSV-1 infection can cause pneumonia in an immunocompetent person. Antiviral treatment using acyclovir is an effective treatment for HSV-1 pneumonitis.
13	19-Year-ole woman was admitted with URI symptoms and required mechanical ventilation for hypoxemia. She was initially treated with broad spectrum antibiotics but showed no improvement. The patient was diagnosed with HSV-1 pneumonia based on cytological and serum studies, treated with acyclovir, and showed substantial improvement overnight.	HSV-1 can cause pneumonia in an immunocompetent person by reactivation of latent HSV infection. Antiviral treatment using acyclovir is effective.

Abbreviations: HSV, herpes simplex virus; PCR, polymerase chain reaction; IV, intravenous; URI, upper respiratory infection.

**Table 2.** Microbiological Studies in Immunocompetent Patients.

Reference No.	Infection at Other Sites	Culture	PCR	DFA	Cytopathology	Bronchoscopic Evaluation	Outcome
9	NR	Negative for virus, fungi, bacteria	Positive for HSV-1	NR	NR	Autopsy-diffuse organizing alveolar damage with focal necrotizing pneumonia	Death
10	NR	Viral and blood cultures negative. Sputum culture positive for *Pseudomonas*.	Serum HSV-1 antibody test positive	NR	Cowdry type A inclusions	Ulcerated vesicles on left bronchus	Patient successfully treated with acyclovir
11	NR	Bronchial cultures positive for *Strep viridans* and *Fusobacterium*. Viral cultures positive for HSV-1.	Positive for HSV-1	NR	Cowdry type A inclusions	Whitish membrane at the level of trachea that bled easily	Patient successfully treated with acyclovir
12	Lips	Negative blood and fungal cultures	Positive for HSV-1. Negative for HSV-2.	Positive for HSV-1 and HSV-2	Cowdry type B inclusion bodies. Tzanck cells present in bronchial scrapings.	Inflamed mucosa in the airways of the right lung	Patient successfully treated with acyclovir
13	NR	Sputum cultures positive for alpha-*Streptococcus* and *Neisseria*. Blood cultures negative.	Serum antibody test positive for HSV-1 and negative for HSV-2	NR	Cowdry type A inclusions	No edema or ulceration noted	Patient successfully treated with acyclovir

Abbreviations: HSV, herpes simplex virus; PCR, polymerase chain reaction; DFA, direct fluorescent antibody; NR, not reported.

He had a second episode of acute respiratory failure requiring mechanical ventilation, and this represented the development of organizing pneumonia. The lung biopsy revealed both organizing pneumonia and diffuse alveolar damage; it did not reveal fungal or mycobacterial infection. Organizing pneumonia is characterized by intra-alveolar fibroblasts mixed with collagen and is usually reversible with corticosteroids.^14^ The process starts with alveolar epithelial damage and necrosis of the pneumocytes, gap formation in the injured epithelial basal laminae, and capillary endothelial injury. As a result of the increased permeability, plasma proteins, including coagulation factors, accumulate in the lumen of the alveoli, and fibrin deposits accumulate in alveolar and bronchiolar spaces. Inflammatory cells and fibroblasts migrate into alveolar spaces, and the latter cells differentiate to myofibroblasts. The final pathology includes fibroblasts and myofibroblasts mixed with connective tissue rich in collagen. This process is often reversible. Pathological changes similar to organizing pneumonia develop after the intranasal inoculation of reovirus in a susceptible strain of mice.^15-17^ This suggests that genetic background may be a factor contributing to the pathogenesis of the condition in mice. Depletion of T cells inhibited the development of fibrosis in this model. In addition, corticosteroids prevented the development of fibrosis when administered early in infection and promoted resolution when administered late in the infection. Diffuse alveolar damage with hyaline membrane formation occurred in the same model when higher doses of virus were used. We assume that herpes infection in our patient injured lung parenchyma and triggered the development of organizing pneumonia. The patient’s pulmonary disease did respond to steroid treatment which supports the diagnosis of organizing pneumonia. Cunha has reported a case of bronchiolitis obliterans and organizing pneumonia in a patient with a renal transplant on tacrolimus who developed herpes pneumonia.^18^

In summary, our patient had no chronic diseases or immunosuppression. He developed an acute pneumonia that appeared to be caused by a herpes simplex type 1. He had a biphasic illness, and during the second phase he developed acute respiratory failure with diffuse pulmonary infiltrates. Open lung biopsy revealed diffuse alveolar damage and organizing pneumonia. The patient responded to corticosteroid treatment and had a good recovery. This case suggests that patients with acute herpes pneumonia can have important complications, including organizing pneumonia, and that patients with organizing pneumonia need more studies to determine whether or not herpes infection contributed to the pathogenesis. This association may have important treatment implications. It is possible that some patients with prolonged mechanical ventilation and herpes infection develop organizing pneumonia, which could contribute to their prolonged intensive care unit course. This development might not be apparent in patients who have serial chest x-rays that show persistent infiltrates throughout the hospital course.

## References

[1] McGinn T Wyer PC Newman TB Keitz S Leipzig R Tips for learners of evidence-based medicine: 3. Measures of observer variability (kappa statistic). CMAJ. 2004;171:1369-1373. 15557592 10.1503/cmaj.1031981 PMC527344

[2] Frisch S Guo AM Diagnostic methods and management strategies of herpes simplex and herpes zoster infections. Clin Geriatr Med. 2013;29:501-526. 23571042 10.1016/j.cger.2013.01.003

[3] Slomka MJ Emery L Munday PE Moulsdale M Brown DWG A comparison of PCR with virus isolation and direct antigen detection for diagnosis and typing of genital herpes. J Med Virol. 1998;55:177-183. 9598940

[4] Ramsey PG Fife KH Hackman RC Meyers JD Corey L Herpes simplex virus pneumonia: clinical, virologic, and pathologic features in 20 patients. Ann Intern Med. 1982;97:813-820. 6293356 10.7326/0003-4819-97-6-813

[5] Luyt CE Combes A Nieszkowska A Trouillet JL Chastre J Viral infections in the ICU. Curr Opin Crit Care. 2008;14:605-608. 18787457 10.1097/MCC.0b013e32830f1e12

[6] Luyt CE Combes A Deback C Herpes simplex virus lung infection in patients undergoing prolonged mechanical ventilation. Am J Respir Crit Care Med. 2007;175:935-942. 17234903 10.1164/rccm.200609-1322OC

[7] van den Brink JW Simoons-Smit AM Beishuizen A Girbes AR Strack van Schijndel RJ Groeneveld AB Respiratory herpes simplex virus type 1 infection/colonisation in the critically ill: marker or mediator? J Clin Virol. 2004;30:68-72. 15072757 10.1016/j.jcv.2003.09.003

[8] Tuxen DV Prevention of lower respiratory herpes simplex virus infection with acyclovir in patients with adult respiratory distress syndrome. Chest. 1994;106(1 suppl):28S-33S. 8020330 10.1378/chest.106.1_supplement.28s

[9] Geradts J Warnock M Yen TS Use of the polymerase chain reaction in the diagnosis of unsuspected herpes simplex viral pneumonia: report of a case. Hum Pathol. 1990;21:118-121. 2153098 10.1016/0046-8177(90)90084-i

[10] Martinez E de Diego A Paradis A Perpiñá M Hernandez M Herpes simplex pneumonia in a young immunocompetent man. Eur Respir J. 1994;7:1185-1188. 7925891

[11] Alvarez-Uria G Surinach JM Ventura A de la Rosa D de Gracia J Fernandez-Sevilla T Herpetic tracheitis and polybacterial pneumonia in an immunocompetent young man is herpes tracheitis involved in the pathogenesis of bacterial pneumonia? J Clin Virol. 2008;41:164-165. 18054276 10.1016/j.jcv.2007.10.014

[12] Hunt DP Muse VV Pitman MB Case records of the Massachusetts General Hospital. Case 12-2013. An 18-year-old woman with pulmonary infiltrates and respiratory failure. N Engl J Med. 2013;368:1537-1545. 23594007 10.1056/NEJMcpc1209608

[13] Reyes CV Bolden JR Herpes simplex virus type-1 pneumonitis in immunocompetent young woman. Heart Lung. 2009;38:526-529. 19944877 10.1016/j.hrtlng.2009.05.002

[14] Cottin V Cordier JF Cryptogenic organizing pneumonia. Semin Respir Crit Care Med. 2012;33:462-475. 23001801 10.1055/s-0032-1325157

[15] Bellum SC Dove D Harley RA Respiratory reovirus 1/L induction of intraluminal fibrosis. A model for the study of bronchiolitis obliterans organizing pneumonia. Am J Pathol. 1997;150:2243-2254. 9176413 PMC1858326

[16] Majeski EI Harley RA Bellum SC London SD London L Differential role for T cells in the development of fibrotic lesions associated with reovirus 1/L-induced bronchiolitis obliterans organizing pneumonia versus Acute Respiratory Distress Syndrome. Am J Respir Cell Mol Biol. 2003;28:208-217. 12540488 10.1165/rcmb.4891

[17] Majeski EI Paintlia MK Lopez AD Harley RA London SD London L Respiratory reovirus 1/L induction of intraluminal fibrosis, a model of bronchiolitis obliterans organizing pneumonia, is dependent on T lymphocytes. Am J Pathol. 2003;163:1467-1479. 14507654 10.1016/S0002-9440(10)63504-3 PMC1868312

[18] Cunha BA Syed U Mickail N Renal transplant with bronchiolitis obliterans organizing pneumonia (BOOP) attributable to tacrolimus and herpes simplex virus (HSV) pneumonia. Heart Lung. 2012;41:310-315. 21996615 10.1016/j.hrtlng.2011.05.009

